# The Supreme Biodegradable Polymer DES in Acute and Chronic Coronary Syndromes: A PIONEER III Substudy

**DOI:** 10.1016/j.jscai.2023.100629

**Published:** 2023-03-27

**Authors:** Yasin Hussain, Shigeru Saito, Michael Curtis, Dean J. Kereiakes, Andreas Baumbach, James P. Zidar, Brent McLaurin, Nabil Dib, Pieter C. Smits, Victor Alfonso Jiménez Díaz, Ángel Cequier, Sjoerd H. Hofma, Cody Pietras, Ovidiu Dressler, M. Ozgu Issever, Stephan Windecker, Martin B. Leon, Alexandra J. Lansky, PIONEER III Trial Investigators

**Affiliations:** a Division of Cardiology, Yale School of Medicine, New Haven, Connecticut; b Shonan Kamakura General Hospital, Kamakura, Japan; c University of Calgary, Alberta, Canada; d The Christ Hospital Heart and Vascular Institute and the Lindner Research Center, Cincinnati, Ohio; e Centre for Cardiovascular Medicine and Devices, William Harvey Research Institute, Queen Mary University of London and Barts Heart Centre, London, United Kingdom; f North Carolina Heart and Vascular, University of North Carolina, Raleigh; g AnMed Health Medical Center, Anderson, South Carolina; h Mercy Gilbert Medical Center, Gilbert, Arizona; i Maasstad Ziekenhuis, Rotterdam, the Netherlands; j Hospital Álvaro Cunqueiro, Vigo, Spain; k Bellvitge Hospital, University of Barcelona, IDIBELL, Barcelona, Spain; l Medisch Centrum Leeuwarden, Hartcentrum Friesland, Leeuwarden, the Netherlands; m Cardiovascular Research Foundation, New York, New York; n Bern University Hospital, Inselspital, University of Bern, Switzerland; o NewYork-Presbyterian Hospital/Columbia University Medical Center, New York, New York

**Keywords:** acute coronary syndromes, biodegradable polymer drug-eluting stent, chronic coronary syndromes, durable polymer drug-eluting stents, endothelium, re-endothelialization

## Abstract

**Background:**

The PIONEER III trial demonstrated noninferiority of 12-month target lesion failure (TLF) with the Supreme DES (Sinomed), a thin-strut cobalt-chromium, biodegradable polymer, sirolimus-eluting stent, compared with a durable polymer, everolimus-eluting (XIENCE/PROMUS) stent (DP-EES). The relative safety and effectiveness of the Supreme DES in patients with acute coronary syndromes (ACS) and those with chronic coronary syndromes (CCS) is not known.

**Methods:**

PIONEER III was a prospective, multicenter, international, 2:1 randomized trial stratified by clinical presentation. The primary end point was TLF at 12 months (a composite of cardiac death, target vessel myocardial infarction, or ischemia-driven target lesion revascularization).

**Results:**

A total of 1628 patients were enrolled, including 41% of patients with ACS (unstable angina and non–ST-elevation myocardial infarction) randomized to Supreme DES (n = 441) versus DP-EES (n = 232) and 59% of patients with CCS randomized to Supreme DES (n = 645) versus DP-EES (n = 310). Patients with ACS were younger, fewer presented with less diabetes, hypertension, and previous revascularization, but more were current smokers. The primary end point of TLF (6.4% vs 4.4%; *P* = .1), major adverse cardiac events (8.5% vs 6.5%; *P* = .16), and stent thrombosis (0.4% vs 0.9%; *P* = .25) at 12 months were similar in the ACS and CCS groups. There was no difference in TLF at 12 months between Supreme DES and DP-EES among patients with ACS (6.6% vs 6.0%; *P* = .89) and those with CCS (4.5% vs 4.3%; *P* = .83); interaction *P* = .51 for TLF by clinical presentation.

**Conclusions:**

Compared with the DP-EES, the Supreme DES seemed safe and effective with a similar TLF at 12 months in both patients with ACS and those with CCS.

## Highlights

• Supreme DES was noninferior to durable polymer EES at 12 months.
• Preclinical studies show Supreme DES achieves early endothelial restoration, which may be of benefit in ACS.
• Supreme DES had similar rates of TLF in patients with ACS and CCS at 12 months.
• Supreme DES had similar rates of TLF to durable-polymer EES irrespective of ACS or CCS presentation.

## Central Illustration

**Figure:**
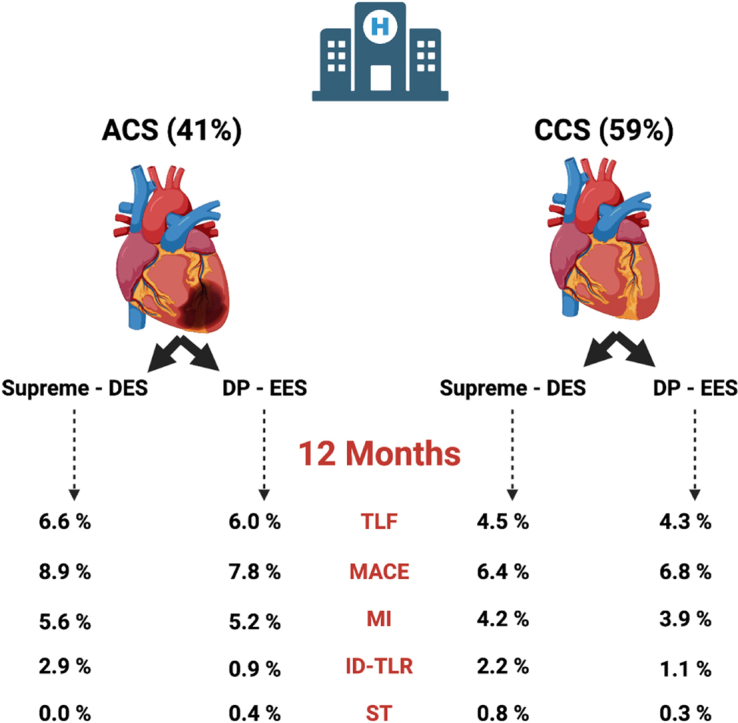

## Introduction

Second-generation drug-eluting stents (DES) are the current standard of care for patients undergoing percutaneous coronary intervention (PCI).^1^, ^2^, ^3^ Patients presenting with acute coronary syndromes (ACS) tend to have worse outcomes than patients with chronic coronary syndromes (CCS).^4^ Evidence from autopsy series of patients treated with DES have demonstrated delayed culprit vessel healing in patients with ACS compared with that in patients with CCS, with less neointimal thickness, greater fibrin deposition, more inflammation, and greater areas of uncovered struts in patients with ACS.^5^ Furthermore, optical coherence tomography (OCT) studies have shown higher rates of incomplete stent apposition and uncovered stent struts in patients with ACS than those in patients with CCS in the short-term and longer term,^6^^,^^7^ and delayed vascular healing has been shown to be an important determinant of stent thrombosis and restenosis.^8^^,^^9^

The Supreme DES (Sinomed) is designed to accelerate early re-endothelialization and vascular healing by delivering the antiproliferative drug sirolimus with simultaneous polymer degradation within an early 4-week to 6-week therapeutic window, leaving behind a stent surface with a biostable ultrathin coating.^10^ Preclinical studies suggest that the Supreme DES achieves early endothelial restoration with improved vascular function and regulation of vascular smooth muscle cell proliferation, which may be of a particular benefit in patients with ACS.^11^, ^12^, ^13^ The PIONEER III trial demonstrated that the Supreme DES was noninferior to durable polymer everolimus-eluting stents (DP-EES) at 12 months.^14^ Whether the safety and effectiveness of the Supreme DES is consistent in patients with ACS compared with those in patients with CCS is not known.

## Methods

### Study design and population

PIONEER III (NCT03168776) is a prospective, 2:1 randomized, single-blind, multicenter trial conducted at 74 investigational sites in North America, Europe, and Japan. Randomization was stratified by ACS presentation. Patients were enrolled from October 2017 to August 2019. We report a prespecified analysis of PIONEER III based on ACS or CCS presentation at the baseline. The rationale, design, inclusion and exclusion criteria, methods, and data management have been reported previously.^14^ In summary, the study included adults with symptomatic CCS with an evidence of ischemia or ACS (unstable angina or non–ST-elevation myocardial infarction [MI]) requiring urgent or elective PCI. The trial was conducted in accordance with the tenets of the Declaration of Helsinki, and all patients provided signed informed consent.

### Study procedures

The Supreme DES is a balloon-expandable, biodegradable polymer (BP), sirolimus-eluting coronary stent system. The stent platform is a laser cut L605 cobalt-chromium alloy tube that is electropolished to a strut thickness of 80.0 μm. Stent struts are covered by a nanometric (∼200.0 nm), nonerodible brush of poly(*n*-butyl methacrylate) that is covalently bonded to the metal surface through a proprietary electrografting (eG coating) process.^15^ The topcoat (3.0-μm to 10.0-μm thick) consists of a poly(lactic-*co*-glycolic acid) BP with sirolimus embedded at a drug density of 1.2 μg/mm^2^; both drug and topcoat are completely resorbed within 4-6 weeks.^16^^,^^17^ The design, safety, and efficacy of the Supreme DES has been extensively characterized.^18^ The control DP-EES (Xience, Abbott Vascular; Promus, Boston Scientific Corporation) is a laser cut cobalt-chromium stent of 81.0-μm strut thickness coated with a 7.8-μm durable fluoride-hexafluoropropylene polymer. The everolimus drug density is 1.0 μg/mm^2^, released by 120 days. PCI was performed according to local standard practices.

All patients were pretreated with aspirin and a P2Y12 inhibitor (clopidogrel, ticagrelor, or prasugrel); dual antiplatelet therapy was continued for at least 6 months and 12 months for patients with ACS.^19^^,^^20^

### End points and outcome measures

The primary end point was the device-oriented outcome of TLF, a composite of cardiac death, target vessel MI, or clinically driven target lesion revascularization (TLR) at 12 months. The secondary end points included the components of the primary end point, death, MI (modified Third Universal Definition^21^), target vessel failure (composite of cardiac death, target vessel MI, or target vessel revascularization), major adverse cardiac events (composite of all-cause death, MI, or target vessel revascularization), bleeding complications defined by the Bleeding Academic Research Consortium,^22^ and stent thrombosis defined by the Academic Research Consortium.^23^ All events were adjudicated by an independent clinical event committee (Cardiovascular Research Foundation), and all baseline angiograms were reviewed by an independent angiographic core laboratory (Yale Cardiovascular Research Group).

### Statistical analysis

Patient randomization was stratified by site and presentation (ACS vs CCS). The PIONEER III trial was designed to demonstrate the noninferiority of the primary TLF end point at 12 months in the intention-to-treat population. There was no prespecified hypothesis in this substudy. We compared patients and outcomes based on presentation (CCS vs ACS) and treatment allocation (Supreme DES vs DP-EES).

Categorical variables are reported as counts and percentages, and comparisons between treatment groups were performed using the χ^2^ or Fisher exact test. Continuous variables are presented as mean and standard deviation and compared with the 2-sample *t* test. If the data failed to meet the assumption for normality per the Shapiro-Wilk test, then the comparisons were made using the Wilcoxon rank sum test. Kaplan-Meier methods were used to calculate time-to-event outcomes, and the log-rank test was used for between-group comparisons. A Cox proportional hazards analysis was used to calculate hazard ratios (HR) with 95% CI and *P* values. Cox proportional hazard assumption was assessed by including a time-dependent covariate (an interaction between the treatment group and logarithm of event time) in the Cox proportional hazard model. A 2-sided *P* < .05 was considered to indicate a statistical significance. SAS version 9.4 (SAS Institute) was used for all statistical analyses.

## Results

The PIONEER III trial enrolled 1628 patients, including 673 (41%) patients with ACS randomized to Supreme DES (n = 441) versus DP-EES (n=232) and 955 (59%) patients with CCS randomized to Supreme DES (n = 645) versus DP-EES (n = 310). Compared with CCS, patients with ACS were younger, fewer presented with less diabetes, hypertension, hyperlipidemia, previous PCI, and previous coronary artery bypass grafting, but more were often previous/current smokers (Table 1). A mean of 1.2 ± 0.5 lesions were treated in both ACS and CCS groups. PCI guidance with a fractional flow reserve (3.6% [30/809] vs 10.6% [123/1136]; *P* < .0001) and intravascular ultrasound (8.4% [69/809] vs 20.0% [231/1136]; *P* < .0001) was used less commonly in patients with ACS. In both ACS and CCS groups, treated lesions were mostly complex, with 64.3% (520/809) versus 67.8% (770/1136) meeting American College of Cardiology/American Heart Association B2/C criteria, respectively (*P* = .12). Patients with ACS presented with fewer lesions with a moderate-to-severe calcification (30.9% [249/807] vs 36.5% [415/1136]; *P* = .014) than patients with CCS, and use of plaque modification was infrequent (<1%) (Table 2). Within the ACS group, patients randomized to Supreme DES recorded a significantly higher percentage of complex lesions (ACC/AHH lesion class B2/C) (66.9% [356/532] vs 59.2% [164/277]; *P* = .04) and significantly a smaller final in-segment minimal lesion diameter (2.57 ± 0.40 mm vs 2.64 ± 0.41 mm; *P* = .03) (Table 2).

**Table 1:** Baseline and procedural characteristics

Characteristics	Acute coronary syndromes				Chronic coronary syndromes				*P*^a^
	All, N = 673	Supreme DES n = 441	DP-EES n = 232	*P*	All, N = 955	Supreme DES, n = 645	DP-EES, n = 310	*P*	
Age, y	62.6 ± 10.5	62.9 ± 10.3	62.0 ± 10.8	.34	65.6 ± 9.4	65.7 ± 9.4	65.4 ± 9.6	.83	<.0001
Female sex	24.2% (163)	21.3% (94)	29.7% (69)	.17	25.4% (243)	25.4% (164)	25.5% (79)	.98	.57
Smoker (current/previous)	64.2% (432)	62.4% (275)	67.7% (157)	.41	58.1% (555)	60.5% (390)	53.2% (165)	.03	<.0001
Diabetes mellitus	25.4% (171)	25.4% (112)	25.4% (59)	.99	33.8% (323)	34.0% (219)	33.5% (104)	.90	.0003
Insulin treatment	33.9% (58)	35.7% (40)	30.5% (18)	.37	33.4% (108/323)	32.4% (71/219)	35.6% (37/104)	.29	.47
Hypertension	68.8% (463)	71.2% (314)	64.2% (149)	.06	75.6% (722)	76.3% (492)	74.2% (230)	.48	.002
Hyperlipidemia	71.9% (484)	72.1% (318)	71.6% (166)	.88	80.2% (766)	80.5% (519)	79.7% (247)	.77	<.0001
Previous MI	15.3% (103)	16.3% (72)	13.4% (31)	.31	19.6% (187)	18.1% (117)	22.6% (70)	.11	.03
Previous PCI	22.7% (153)	23.8% (105)	20.7% (48)	.36	33.2% (317)	30.9% (199)	38.1% (118)	.03	<.0001
Previous CABG	2.8% (19)	3.4% (15)	1.7% (4)	.21	6.0% (57)	5.9% (38)	6.1% (19)	.88	.003
Previous stroke	2.8% (19)	2.5% (11)	3.4% (8)	.48	4.8% (46)	5.4% (35)	3.5% (11)	.20	.043
Renal insufficiency	7.9% (53)	7.9% (35)	7.8% (18)	.74	8.0% (76)	7.9% (51)	8.1% (25)	.93	.95
No. of diseased vessels									
1	68.9% (464)	70.1% (309)	66.8% (155)	.39	73.2% (699)	74.3% (479)	71.0% (220)	.28	.06
2	24.2% (163)	21.8% (96)	28.9% (67)	.04	18.6% (178)	18.1% (117)	19.7% (61)	.57	.006
≥3	6.9% (46)	8.1% (36)	4.3% (10)	.02	8.2% (78)	7.6% (49)	9.3% (29)	.43	.29
Procedural characteristics									
No. of vessels treated per patient	1.14 ± 0.35	1.14 ± 0.36	1.13 ± 0.34	.91	1.11 ± 0.31	1.10 ± 0.29	1.14 ± 0.35	.05	.12
Multiple vessels treated	13.5% (91)	13.6% (60)	13.4% (31)	.94	11.0% (105)	9.6% (62)	13.9% (43)	.05	.17
No. of target lesions per patient	1.2 ± 0.5	1.2 ± 0.5	1.2 ± 0.4	.43	1.2 ± 0.5	1.2 ± 0.4	1.3 ± 0.5	.06	.44
1	79.5% (532/672)	80.5% (355)	77.5% (179/231)	.36	81.3% (776)	82.8% (534)	78.1% (242)	.08	.37
2	18.6% (125/672)	17.0% (75)	21.6% (50/231)	.14	15.8% (151)	15.0% (97)	17.4% (54)	.35	.14
3	1.9% (13/672)	2.5% (11)	0.9% (2/231)	.24	2.9% (28)	2.2% (14)	4.5% (14)	.04	.21
No. of stents per patient	1.3 ± 0.6	1.2 ± 0.6	1.3 ± 0.6	.10	1.2 ± 0.6	1.2 ± 0.6	1.3 ± 0.6	.09	.82
Radial access	85.7% (577)	86.4% (381)	84.5% (196)	.50	76.5% (731)	77.4% (499)	74.8% (232)	.39	<.0001
Brachial access	0.3% (2)	0.2% (1)	0.4% (1)	1.0	0.6% (6)	0.6% (4)	0.6% (2)	1.0	.48
Femoral access	14.0% (94)	13.4% (59)	15.1% (35)	.54	22.8% (218)	22.0% (142)	24.5% (76)	.39	<.0001
Hemostasis device use	76.7% (516)	77.8% (343)	74.6% (173)	.35	71.3% (681)	72.1% (465)	69.7% (216)	.44	.016

Values are given as mean ± standard deviation or % (n).

CCS, chronic coronary syndromes; DES, drug-eluting stent; DP-EES, durable polymer everolimus-eluting stent.

a All patients with acute coronary syndromes (ACS) vs all patients with CCS regardless of stent.

**Table 2:** Angiographic characteristics

	Acute coronary syndromes				Chronic coronary syndromes				*P*^a^
	All, N = 673 (809 lesions)	Supreme DES, n = 441 (532 lesions)	DP-EES, n = 232 (277 lesions)	*P*	All, N = 955 (1136 lesions)	Supreme DES, n = 645 (751 lesions)	DP-EES n = 310 (385 lesions)	*P*	
Stent implantation characteristics									
No. of stents per lesion	1.1 ± 0.3	1.1 ± 0.3	1.1 ± 0.3	.92	1.1 ± 0.3	1.1 ± 0.3	1.0 ± 0.2	.12	.43
Stented lesion length, mm	22.26 ± 9.43	22.57 ± 9.65	21.66 ± 8.99	.12	22.14 ± 8.51	22.30 ± 8.67	21.81 ± 8.19	.22	.62
Maximum stent diameter, mm	2.97 ± 0.43	2.95 ± 0.42	3.00 ± 0.44	.24	2.91 ± 0.41	2.91 ± 0.41	2.91 ± 0.42	.61	.004
Procedural characteristics									
Fractional flow reserve	3.6% (30)	3.7% (20)	3.5% (10)	.88	10.6% (123)	10.2% (78)	11.5% (45)	.49	<.0001
Intravascular ultrasound	8.4% (69)	8.9% (48)	7.3% (21)	.44	20.0% (231)	19.9% (152)	20.2% (79)	.89	<.0001
Predilation	76.4% (630)	79.6% (429)	70.3% (201)	.03	75.9% (887)	77.5% (593)	72.6% (284)	.07	.80
Postdilation	53.6% (440)	53.2% (285)	54.4% (155)	.74	51.5% (591)	51.7% (393/760)	51.0% (198/388)	.83	.35
Target vessel location^b^									
Left anterior descending	43.3% (350)	44.5% (237)	40.8% (113)	.18	46.0% (522)	45.9% (345)	46.0% (177)	.94	.24
Left circumflex	26.6% (215)	27.1% (144)	25.6% (71)	.96	24.4% (227)	25.3% (190)	22.6% (87)	.38	.28
Right coronary	30.2% (244)	28.4% (151)	33.6% (93)	.13	29.6% (236)	28.6% (215)	31.4% (121)	.34	.77
Left main	0.0% (0)	0.0% (0)	0.0% (0)	NA	0.1% (1)	0.1% (1)	0.0% (0)	1.0	.98
ACC/AHA lesion class									
A	9.4% (76)	8.6% (46)	10.8% (30)	.33	7.7% (88)	7.1% (53)	9.1% (35)	.23	.20
B1	26.3% (213)	24.4% (130)	30.0% (83)	.11	24.5% (278)	25.8% (194)	21.8% (84)	.14	.37
B2	26.1% (211)	27.3% (145)	23.8% (66)	.30	28.2% (320)	26.2% (197)	31.9% (123)	.05	.32
C	38.2% (309)	39.7% (211)	35.4% (98)	.28	39.6% (450)	40.9% (307)	37.1% (143)	.24	.53
B2/C	64.3% (520)	66.9% (356)	59.2% (164)	.04	67.8% (770)	67.1% (504)	69.1% (266)	.50	.12
Calcification (moderate/severe)	30.9% (249/807)	31.7% (168/530)	29.2% (81)	.56	36.5% (415)	37.0% (278)	35.6% (137)	.77	.014
Any bifurcation	21.1% (171)	21.4% (114)	29.6% (57)	.78	22.3% (253)	22.4% (168)	22.1% (85)	.94	.55
Baseline QCA results^b^									
Reference diameter, mm	2.74 ± 0.45	2.72 ± 0.44	2.77 ± 0.47	.10	2.68 ± 0.48	2.69 ± 0.48	2.67 ± 0.47	.47	.01
Minimal lumen diameter, mm	0.87 ± 0.38	0.85 ± 0.37	0.90 ± 0.40	.07	0.97 ± 0.39	0.98 ± 0.40	0.96 ± 0.39	.40	<.0001
Percent diameter stenosis, %	68.02 ± 13.04	68.35 ± 12.97	67.37 ± 13.16	.31	63.63 ± 13.19	63.49 ± 13.19	63.92 ± 13.20	.68	<.0001
Lesion length, mm^c^	15.22 ± 7.73	15.53 ± 8.16	14.64 ± 6.83	.16	15.06 ± 7.13	15.11 ± 7.10	14.95 ± 7.20	.73	.63
Final QCA result^b^									
In-stent minimal lumen diameter, mm^c^	2.70 ± 0.40	2.68 ± 0.40	2.73 ± 0.40	.09	2.65 ± 0.40	2.65 ± 0.41	2.65 ± 0.38	.96	.012
In-stent diameter stenosis, %^c^	7.99 ± 4.72	8.14 ± 4.97	7.70 ± 4.22	.20	8.38 ± 4.58	8.50 ± 4.34	8.14 ± 5.01	.21	.07
In-stent acute gain, mm^c^	1.83 ± 0.46	1.83 ± 0.46	1.83 ± 0.47	.92	1.68 ± 0.45	1.67 ± 0.45	1.69 ± 0.44	.56	<.0001
Segment minimal lumen diameter, mm^d^	2.59 ± 0.41	2.57 ± 0.40	2.64 ± 0.41	.03	2.55 ± 0.43	2.55 ± 0.43	2.54 ± 0.42	.74	.02
Segment diameter stenosis, %^d^	9.65 ± 3.86	9.70 ± 3.89	9.55 ± 3.81	.66	10.06 ± 4.80	10.09 ± 4.86	10.00 ± 4.68	.75	.06
Segment acute gain, mm^d^	1.72 ± 0.47	1.72 ± 0.48	1.73 ± 0.47	.69	1.57 ± 0.46	1.57 ± 0.46	1.58 ± 0.46	.82	<.0001

Values are given as mean ± standard deviation or % (n).

ACC, American College of Cardiology; AHA, American Heart Association; QCA, quantitative coronary angiography.

a All patients with acute coronary syndromes (ACS) vs all patients with chronic coronary syndromes (CCS) regardless of stent.

b The results reported based on the angiographic core laboratory analysis.

c The total number of lesions was 802 for ACS and 1120 for CCS.

d The total number of lesions was 804 for ACS and 1127 for CCS.

**Figure 1:**
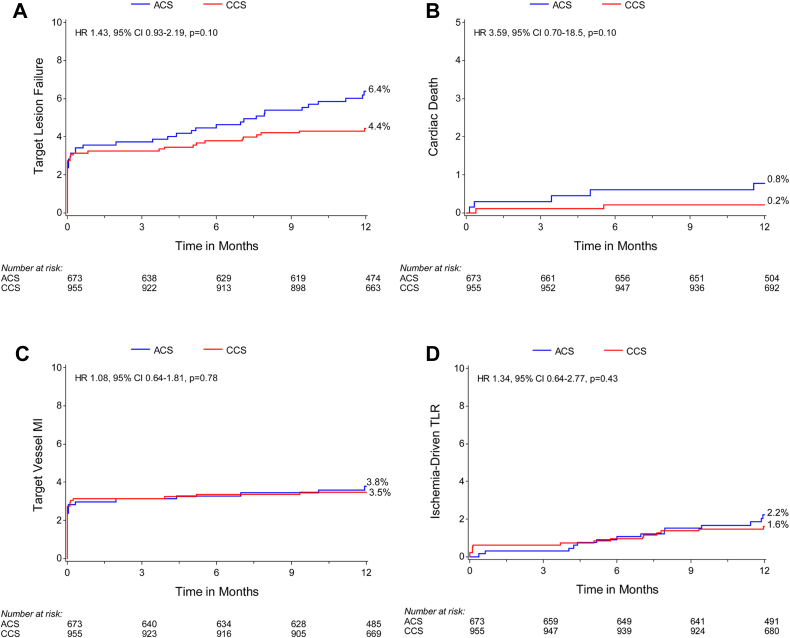
**Kaplan-Meier time-to-event curves for target lesion failure and its components in patients with ACS versus CCS.** (A) Primary outcome (target lesion failure); (B) cardiac death; (C) target vessel MI; (D) clinically driven target lesion revascularization (TLR). ACS, acute coronary syndromes; CCS, chronic coronary syndromes; HR, hazard ratio; MI, myocardial infarction.

**Figure 2:**
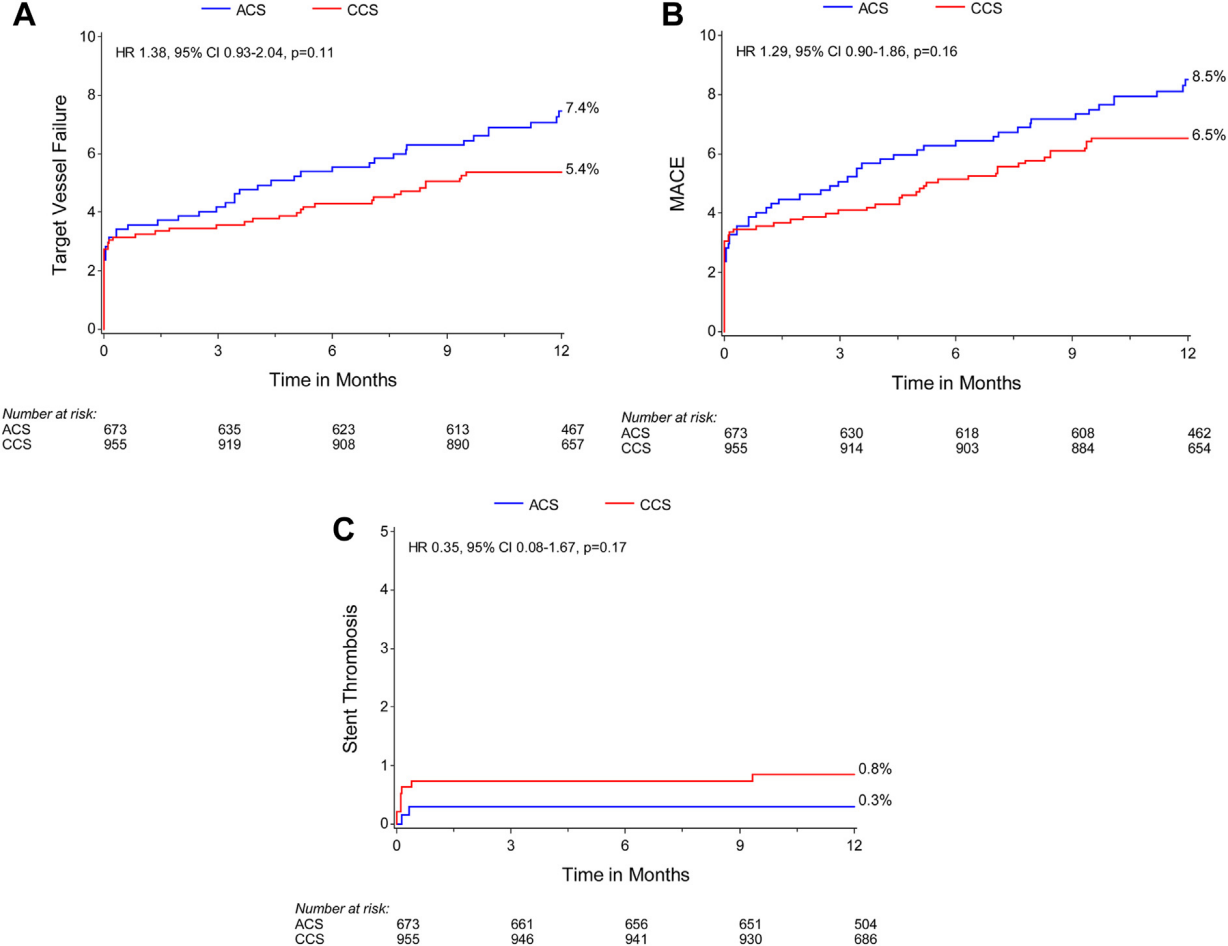
**Kaplan-Meier time-to-event curves for secondary outcomes in patients with ACS versus CCS.** (A) Target vessel failure; (B) major adverse cardiac events (MACE); (C) stent thrombosis (definite and probable). ACS, acute coronary syndromes; CCS, chronic coronary syndromes; HR, hazard ratio.

At 12 months, there was not sufficient evidence to determine a difference in the primary end point of TLF (6.4% [42/673] vs 4.4% [42/955]; HR, 1.43; 95% CI, 0.93-2.19; *P* = .10) (Supplemental Table S1 and Figure 1A) or the components of the primary outcome including cardiac death, target vessel MI, and clinically driven TLR and target vessel failure, major adverse cardiac events, and definite or probable stent thrombosis were similar between the ACS and CCS groups (Figures 1B-D and 2). The rates of minor and major bleeding (4.2% [28/673] vs 2.1% [20/955]; HR, 2.02; 95% CI, 1.14-3.59; *P* = .014) and spontaneous MI (3.0% [19/673] vs 1.3% [12/955]; HR, 2.27; 95% CI, 1.10-4.68; *P* = .02) were higher in patients with ACS than those in patients with CCS (Supplemental Table S1).

**Figure 3:**
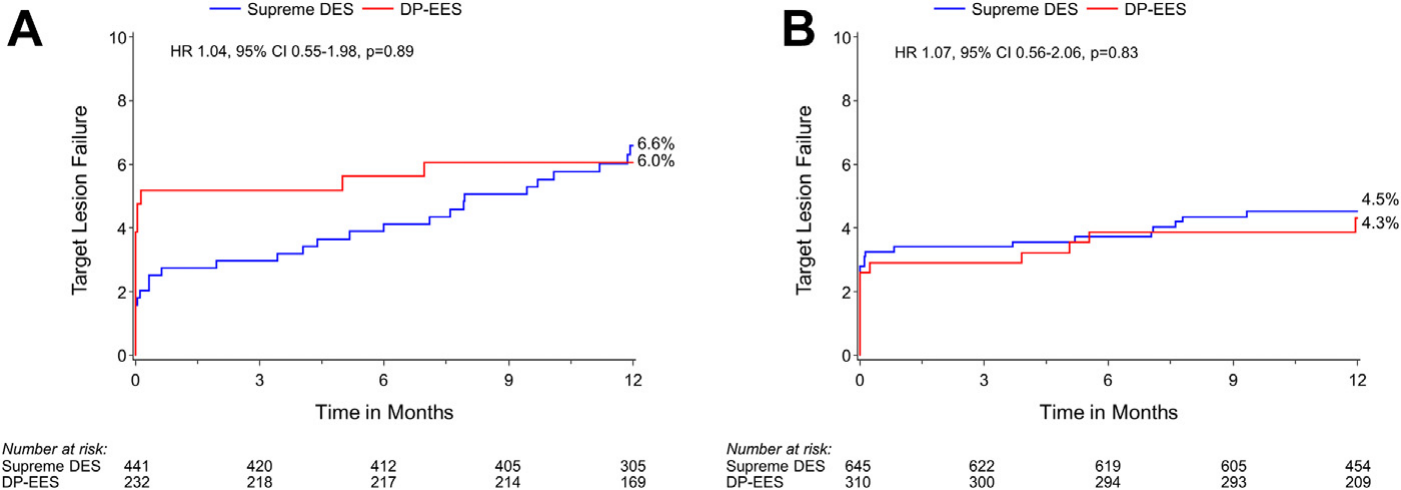
**Kaplan-Meier time-to-event curves for target lesion failure in patients with ACS and CCS based on stent type.** (A) Target vessel failure in ACS; (B) target vessel failure in CCS. ACS, acute coronary syndromes; CCS, chronic coronary syndromes; DES, drug-eluting stents; DP-EES, durable polymer everolimus-eluting stents; HR, hazard ratio.

TLF at 12 months was similar for the Supreme DES and DP-EES subgroups among patients with ACS (6.6% [28/441] vs 6.0% [14/232]; *P* = .89) and those with CCS (4.5% [29/645] vs 4.3% [13/310]; *P* = .83) (Figure 3 and Central Illustration); the interaction *P* = .51 for TLF by clinical presentation. Stent thrombosis in patients with ACS occurred in 0.2% (1/441) and 0.4% (1/232) (*P* = .64) of patients with Supreme DES and DP-EES respectively. In patients with CCS, stent thrombosis occurred in 1.1% (7/645) and 0.3% (1/310) (*P* = .23) of patients with Supreme DES and DP-EES, respectively. In the ACS group, periprocedural MI was higher in the DP-EES group (1.6% [7/441] vs 4.7% [11/232]; *P* = .02), but spontaneous MI was higher in the Supreme DES group (4.0% [17/441] vs 1.0% [2/232]; *P* = .03) (Table 3).

**Central Illustration:**
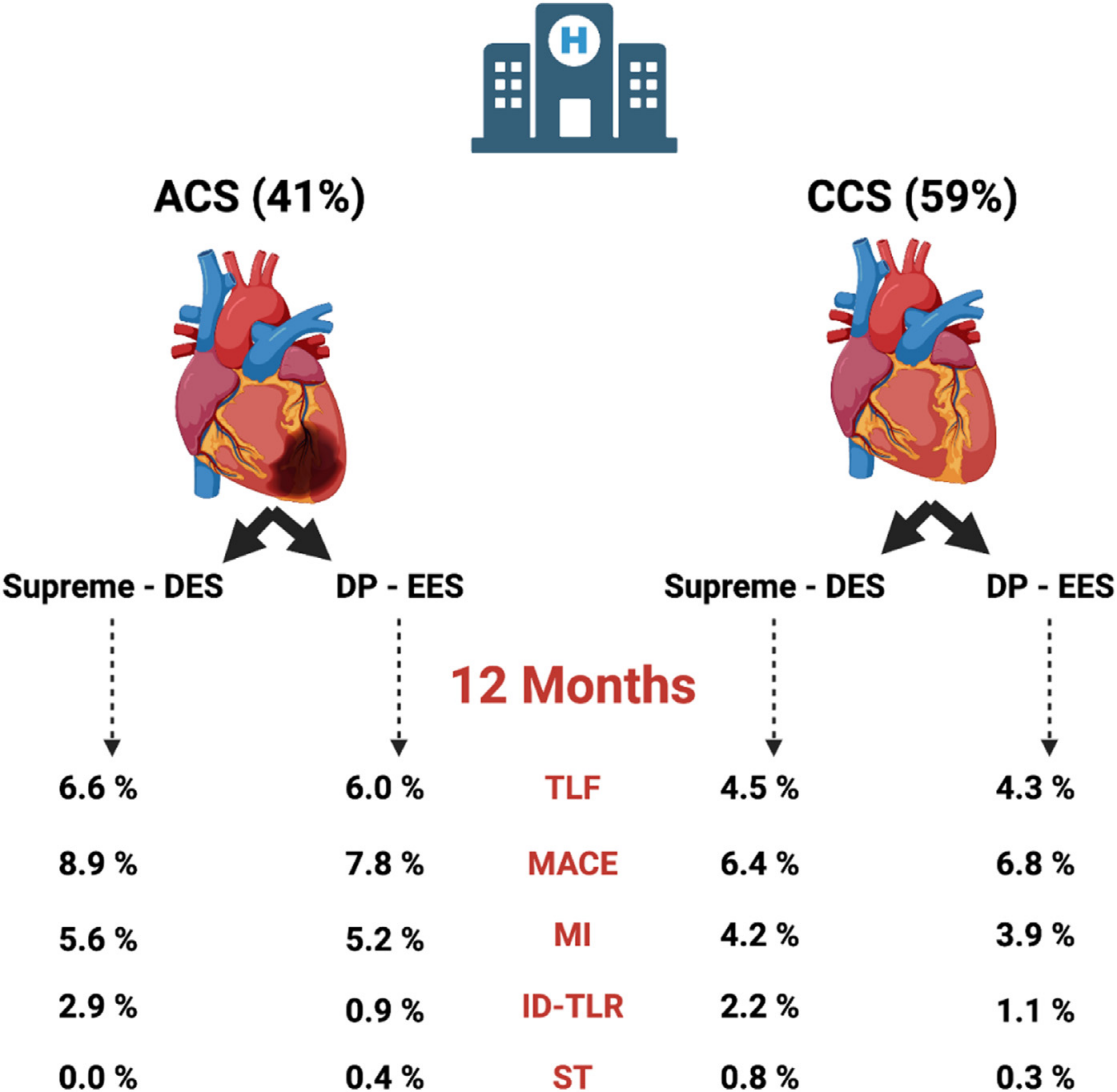
**Primary and secondary endpoints at 12 months in Supreme drug-eluting stent (DES) and durable polymer everolimus-eluting stent (DP-EES) in both acute coronary syndromes (ACS) and chronic coronary syndromes (CCS) presentation.** ID-TLR, ischemia-driven target lesion revascularization; MACE, major adverse cardiac events; MI, myocardial infarction; ST, stent thrombosis; TLF, target lesion failure.

**Table 3:** Outcomes at 1 year by presentation and treatment

	Acute coronary syndromes			Chronic coronary syndromes			*P*_interaction_
	Supreme DES, n = 441	DP-EES, n = 232	*P*	Supreme DES, n = 645	DP-EES, n = 310	*P*	
Primary outcome							
Target lesion failure	6.6% (28)	6.0% (14)	.89	4.5% (29)	4.3% (13)	.83	.96
Secondary outcomes							
Major adverse coronary events	8.9% (38)	7.8% (18)	.72	6.4% (41)	6.8% (21)	.81	.67
Target vessel failure	7.3% (31)	7.8% (18)	.72	5.5% (35)	5.2% (16)	.86	.71
Any death	0.7% (3)	1.8% (4)	.21	0.5% (3)	1.3% (4)	.16	.93
Cardiac death	0.5% (2)	1.4% (3)	.43	0.2% (1)	0.3% (1)	.59	.86
Any myocardial infarction	5.6% (24)	5.2% (13)	.90	4.2% (27)	3.9% (12)	.82	.95
Periprocedural	1.6% (7)	4.7% (11)	.02	3.1% (20)	2.9% (9)	.87	.06
Spontaneous	4.0% (17)	1.0% (2)	.03	1.2% (8)	1.3% (4)	.95	.11
Any revascularization	6.4% (28)	3.6% (8)	.11	4.2% (27)	4.3% (13)	1.00	.26
Target lesion revascularization	2.9% (12)	0.9% (2)	.10	2.2% (14)	1.1% (3)	.19	.72
Clinically driven	2.9% (12)	0.9% (2)	.10	1.9% (12)	1.1% (3)	.30	.61
Target vessel revascularization	4.5% (19)	3.1% (7)	.40	3.3% (21)	2.9% (9)	.77	.67
Clinically driven	4.5% (20)	3.1% (7)	.40	3.0% (19)	2.9% (9)	.97	.55
Nontarget vessel revascularization	3.0% (13)	1.8% (4)	.33	1.6% (10)	2.1% (6)	.66	.37
Any bleeding (BARC definition)	3.5% (15)	5.7% (13)	.17	2.5% (16)	1.3% (4)	.23	.08
BARC 3 or 5	3.3% (14)	1.3% (3)	.14	1.6% (10)	0.7% (2)	.24	.97
Definite stent thrombosis	0.0%	0.4% (1)	.17	0.9% (6)	0.3% (1)	.30	.99
Early (0-30 d)	0.0%	0.4% (1)	.17	0.8% (5)	0.3% (1)	.41	.99
Late (31-365 d)	0.0%	0.0%	—	0.2% (1)	0.0%	.49	>.99
Definite/probable stent thrombosis	0.2% (1)	0.4% (1)	.64	1.1% (7)	0.3% (1)	.23	.30
Early (0-30 d)	0.2% (1)	0.4% (1)	.64	0.9% (6)	0.3% (1)	.30	.34
Late (31-360 d)	0.0%	0.0%	—	0.2% (1)	0.0%	.49	>.99

Values are given as % (n).

BARC, Bleeding Academic Research Consortium; DES, drug-eluting stent; DP-EES, durable polymer everolimus-eluting stent.

## Discussion

This substudy of the PIONEER III trial stratified by clinical presentation shows the following important observations: (1) the primary device-specific outcome of TLF and its components were similar in the ACS and CCS groups at 12 months; (2) patients with ACS had more periprocedural bleeding and spontaneous MI; and (3) Supreme DES had similar rates of TLF with DP-EES irrespective of ACS or CCS presentation.

### Outcomes based on ACS and CCS presentations

Unlike previous studies, this substudy did not demonstrate significant differences in outcomes between the ACS and CCS groups. Although patients with ACS showed increased platelet activation and delayed vessel healing compared with patients with CCS,^5^^,^^24^ the lower risk profile of the ACS cohort likely balances the overall observed outcomes of the ACS and CCS groups. In the PIONEER III trial, patients with ACS showed a significantly lower comorbid disease than those with CCS and were younger, with fewer presenting with diabetes, hypertension, hyperlipidemia, previous revascularization, and less lesions with moderate-to-severe calcification, all of which likely contribute to worse clinical outcomes for patients with CCS.^25^ Furthermore, although periprocedural MI may be more difficult to detect in ACS than that in CCS, we did observe a higher risk of spontaneous MI among patients with ACS which is in line with other studies.^26^^,^^27^ It is important to note that the study excluded patients with ST-elevation myocardial infarction (STEMI), who have been shown to experience a higher mortality within the first year of intervention than patients with other ACS.^28^

### Device-related outcomes based on ACS and CCS presentations

Vascular response to stent implantation in patients with ACS has been shown to be associated with a less neointimal thickness, greater fibrin deposition, more inflammation, and greater areas of uncovered struts in autopsy series,^5^ and OCT studies have confirmed a greater stent malapposition and more uncovered struts in patients with ACS.^7^ Furthermore, perfusion imaging studies have shown that patients with ACS show significantly impaired vasodilator response compared with patients with CCS after PCI, and this impairment persists as long as 6 months.^29^ Immunohistologic studies investigating circulating endothelial cells, which are associated with endothelial injury, show significantly higher circulating endothelial cells counts in ACS than those in CCS.^30^ Because the endothelium plays a central role in releasing vasoactive substances and is the main modulator of vascular tone,^31^ these studies suggest that, in ACS, endothelial injury is accentuated, and its function is impaired long after the acute insult.

The Supreme DES design emphasizes early synchronized elution of antiproliferative drug and polymer degradation to promote early endothelial restoration.^14^ The concept that the Supreme DES achieves early healing and restoration of endothelial function is supported by intracoronary OCT data from a randomized study, showing more complete strut coverage at 1 month than that with DP-DES (83.8 ± 10.4% vs 73.0 ± 17.5%; *P* = .04).^32^ Furthermore, in vivo data using Evans Blue staining and P120/VE-cadherin co-staining to assess the integrity of the endothelial barrier showed an improved restoration of endothelial function with Supreme DES compared with that with other second-generation DESs.^33^ The results from the PIONEER III trial show that device-specific outcomes with Supreme DES, such as TLF and ST, were similar to DP-EES for both ACS and CCS groups at 12 months. Given that the PIONEER III trial was designed to demonstrate the noninferiority of TLF between devices at 1 year and differentiation in outcomes with Supreme would be expected after 1 year, the current results with Supreme DES are reassuring, especially in the ACS cohort. Among patients with ACS, clinically driven TLR was numerically higher and spontaneous MI was significantly higher in the Supreme DES group than those in the DP-EES group at 12 months, which may be due to an imbalance of patients with more complex lesion (American College of Cardiology/American Heart Association class B2/C) and smaller final segment minimal lesion diameter at the end of the procedure in patients with ACS randomized to the Supreme DES group, both factors known to be associated with worse clinical outcomes such as MI and TLR.^34^^,^^35^ The higher observed rate of spontaneous MI in the Supreme group was likely related to nontarget lesion plaque rupture rather than stent thrombosis related to the Supreme DES because adjudicated rates of stent thrombosis were exceedingly rare in both groups. In the ACS population, periprocedural MI occurred more frequently in the DP-EES population, with the prognostic importance of periprocedural MI being substantially less than that of spontaneous MI.^26^

Several studies have evaluated biodegradable polymer drug-eluting stents (BP-DESs) in patients with ACS and yielded mostly modest or no benefit, and outcomes vary based on specific stent designs.^36^ The only BP-DES that has shown consistent benefit in ACS populations is the ultrathin bioresorbable Orsiro stent. The BIOFLOW V trial included 677 patients with ACS, in whom the Orsiro BP-DES placement resulted in a reduction in TLF at 12 months compared with the Xience DP-EES placement (5.6% vs 11.0%; difference, −5.37%; 95% CI, −10.66% to −0.96%; *P* = .02).^37^ In a subgroup analysis of the BIOSCIENCE trial including patients with STEMI, the Orsiro stent reduced TLF at 12 months compared with Xience (3.4% vs 8.8%; rate ratio [RR], 0.38; 95% CI, 0.16-0.91; *P* = .02),^38^ although the results were no longer significant at 5 years (16.9% vs 16.0%; RR, 1.04; 95% CI, 0.78-1.41; *P* = .78).^39^ In the BIOSTEMI trial of patients with STEMI exclusively, the Orsiro stent resulted in a significant reduction in TLF compared with Xience (4.0% vs 6.0%; RR, 0.59; 95% Bayesian CrI, 0.37-0.94).^40^ Orsiro is not only the alone DES showing superiority in patients with STEMI but also the alone DES to show an inferior performance in treating complex calcified lesions.^41^ Studies of other BP-DES in patients with ACS have not demonstrated clinical benefit compared with DP-DES,^42^^,^^43^ highlighting that not all BP-DES are created equal, and each DES needs to be evaluated individually because there is no evidence for a class effect of BP-DESs.

### Limitations

The PIONEER III trial was designed to demonstrate the noninferiority of the primary composite TLF end point. Although randomization was stratified by ACS presentation, this subgroup analysis was not powered to demonstrate the noninferiority or differences in event rates between ACS and CCS populations or treatment groups. Therefore, the results are purely hypothesis generating. PIONEER III excluded patients with STEMI, and multivessel and multilesion treatment were uncommon, and the results only apply to the population studied.

## Conclusions

In the PIONEER III trial, there was no sufficient evidence to suggest a difference in TLF at 12 months between Supreme DES and DP-EES in both ACS and CCS groups. Ongoing follow-up to 5 years will determine whether a clinical benefit exists in the longer term.
