# Supplementary material for: The Supreme Biodegradable Polymer DES in Acute and Chronic Coronary Syndromes: A PIONEER III Substudy
Source: J Soc Cardiovasc Angiogr Interv. 2023 Mar 27;2(3):100629. doi: 10.1016/j.jscai.2023.100629 (PMC11307614; doi:10.1016/j.jscai.2023.100629)
Supplement: Supplemental Table S1 [file mmc1.docx]

**Supplemental Table 1. Outcomes at 1 Year by Presentation**

|  | **Acute Coronary Syndromes n=673** | **Chronic Coronary Syndromes n=955** | **Hazard Ratio (95% Confidence Interval)** | **p Value** |
| --- | --- | --- | --- | --- |
| Primary outcome |  |  |  |  |
| Target lesion failure | 6.4 | 4.4 | 1.43 (0.93-2.19) | 0.10 |
| Secondary outcomes |  |  |  |  |
| Major adverse cardiac events | 8.5 | 6.5 | 1.30 (0.90-1.86) | 0.16 |
| Target vessel failure | 7.4 | 5.4 | 1.38 (0.93-2.04) | 0.11 |
| Any death | 1.1 | 0.7 | 1.44 (0.50-4.10) | 0.50 |
| Cardiac death | 0.8 | 0.2 | 3.59 (0.70-18.49) | 0.10 |
| Any myocardial infarction | 5.5 | 4.1 | 1.32 (0.84-2.07) | 0.23 |
| Periprocedural | 2.7 | 3.0 | 0.88 (0.49-1.59) | 0.67 |
| Spontaneous | 3.0 | 1.3 | 2.27 (1.10-4.68) | 0.02 |
| Any revascularization | 5.6 | 4.2 | 1.30 (0.83-2.04) | 0.25 |
| Target lesion revascularization | 2.2 | 1.8 | 1.18 (0.58-2.39) | 0.65 |
| Clinically driven | 2.2 | 1.6 | 1.34 (0.64-2.77) | 0.43 |
| Target vessel revascularization | 4.0 | 3.2 | 1.24 (0.74-2.10) | 0.41 |
| Clinically driven | 4.0 | 3.0 | 1.34 (0.78-2.28) | 0.29 |
| Non-target vessel revascularization | 2.6 | 1.7 | 1.54 (0.78-3.04) | 0.21 |
| Any bleeding (BARC definition) | 4.2 | 2.1 | 2.02 (1.14-3.59) | 0.014 |
| BARC 3 or 5 | 2.6 | 1.3 | 2.04 (0.97-4.27) | 0.054 |
| Definite stent thrombosis | 0.1 | 0.7 | 0.20 (0.02-1.65) | 0.10 |
| Early (0-30 day) | 0.1 | 0.6 | 0.24 (0.03-1.96) | 0.15 |
| Late (31-365 days) | 0.0 | 0.1 | — | 0.40 |
| Definite/probable stent thrombosis | 0.3 | 0.8 | 0.35 (0.08-1.67) | 0.17 |
| Early (0-30 day) | 0.3 | 0.7 | 0.40 (0.08-1.95) | 0.24 |
| Late (31-360 days) | 0.0 | 0.1 | — | 0.40 |

Values are %. BARC = Bleeding Academic Research Consortium.
